# Serosurveillance and Molecular Investigation of Wild Deer in Australia Reveals Seroprevalence of *Pestivirus* Infection

**DOI:** 10.3390/v12070752

**Published:** 2020-07-13

**Authors:** Jose L. Huaman, Carlo Pacioni, David M. Forsyth, Anthony Pople, Jordan O. Hampton, Teresa G. Carvalho, Karla J. Helbig

**Affiliations:** 1 Department of Physiology, Molecular Virology Laboratory, Anatomy and Microbiology, School of Life Sciences, La Trobe University, Melbourne 3086, Australia; j.huamantorres@latrobe.edu.au; 2 Department of Physiology, Molecular Parasitology Laboratory, Anatomy and Microbiology, School of Life Sciences, La Trobe University, Melbourne 3086, Australia; t.carvalho@latrobe.edu.au; 3 Department of Environment, Land, Water and Planning, Arthur Rylah Institute for Environmental Research, Heidelberg 3084, Australia; carlo.pacioni@delwp.vic.gov.au; 4 School of Veterinary and Life Sciences, Murdoch University, South Street, Murdoch, WA 6150, Australia; jordan.hampton@murdoch.edu.au; 5 NSW Department of Primary Industries, Vertebrate Pest Research Unit, Orange 2800, Australia; dave.forsyth@dpi.nsw.gov.au; 6 Department of Agriculture and Fisheries, Invasive Plants & Animals Research, Biosecurity Queensland, Ecosciences Precinct, Brisbane 4102, Australia; tony.pople@daf.qld.gov.au; 7 Ecotone Wildlife, P.O. Box 76, Inverloch, VIC 3996, Australia

**Keywords:** Australia, deer, prevalence, *Pestivirus*, ruminants, serosurveillance, virology, wildlife disease

## Abstract

Since deer were introduced into Australia in the mid-1800s, their wild populations have increased in size and distribution, posing a potential risk to the livestock industry, through their role in pathogen transmission cycles. In comparison to livestock, there are limited data on viral infections in all wildlife, including deer. The aim of this study was to assess blood samples from wild Australian deer for serological evidence of exposure to relevant viral livestock diseases. Blood samples collected across eastern Australia were tested by ELISA to detect antigens and antibodies against *Pestivirus* and antibodies against bovine herpesvirus 1. A subset of samples was also assessed by RT-PCR for *Pestivirus*, Simbu serogroup, epizootic hemorrhagic disease virus and bovine ephemeral fever virus. Our findings demonstrated a very low seroprevalence (3%) for ruminant *Pestivirus*, and none of the other viruses tested were detected. These results suggest that wild deer may currently be an incidental spill-over host (rather than a reservoir host) for *Pestivirus*. However, deer could be a future source of viral infections for domestic animals in Australia. Further investigations are needed to monitor pathogen activity and quantify possible future infectious disease impacts of wild deer on the Australian livestock industry.

## 1. Introduction

Deer (family Cervidae) often attain high densities when introduced to new areas [1]. As cervids are ungulates and closely related to economically important livestock species including cattle, sheep and goats, it is unsurprising that they share many pathogens, including several of major agricultural relevance. Many viral pathogens of farm ruminants have been detected in wild cervids globally, the most important of which are bovine viral diarrhea virus (BVDV), bovine herpesvirus 1 (BoHV-1), epizootic hemorrhagic disease virus (EHDV) and bovine ephemeral fever virus (BEFV) [2,3,4,5,6].

In Australia, wild populations of six non-native deer species became established in the late 1800s and early 1900s. These populations have expanded in abundance and distribution [1,2]. Wild deer in Australia commonly share grazing areas with livestock, and their susceptibility to a wide range of viral infections of importance to the livestock industry has been demonstrated [2]. Hence, wild deer represent a significant potential source of pathogen transmission to livestock [1].

Australian agriculture is currently free from many viral diseases that impact livestock industries in other parts of the world, including foot-and-mouth disease (FMD) and louping ill virus. However, the issue of cervid-transmitted disease in Australia is important because incursions or outbreaks of emerging, exotic or endemic disease could cause serious production losses, resulting in substantial economic impacts for the livestock industry [1,2]. Transmission of disease by cervids could also prevent effective control, management or eradication of a livestock disease, resulting in prolonged epidemics [2].

There is currently limited information about the infection status of Australia’s wild deer populations. A small number of studies assessed the distribution of BVDV in wild fallow (*Dama dama*) and red (*Cervus elaphus*) deer populations in the 1970s and 1980s [7,8], but these were conducted in small geographical areas. Moreover, serological studies of endemic livestock viruses including BEFV, EHDV and Simbu serogroup virus were performed in red deer from Queensland [9] and rusa deer (*Rusa timorensis*) from New South Wales [10]. Therefore, the role wild deer might play in the spread of diseases to livestock remains unclear. Addressing this knowledge gap is important for anticipating how viruses might be transmitted to other animals, and how diseases might be controlled. The objective of this study was to assess whether Australian wild deer populations are exposed to relevant viral livestock diseases by testing blood samples for antibodies and antigens or through detection of viral genetic material. Our results establish a baseline exposure level, and possible spreading patterns in Australia’s wild deer.

## 2. Materials and Methods

### 2.1. Sampling

**Figure 1:**
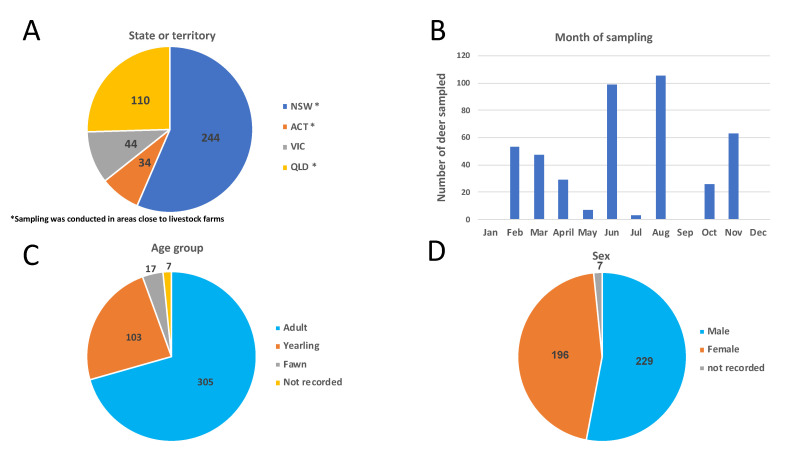
Population characteristics and distribution of deer sampled and tested in this study. Total numbers of deer sampled per region (**A**), per month (**B**), by age group (**C**) and by sex (**D**) are represented graphically.

Blood samples were collected by recreational hunters and professional culling staff from deer populations across eastern Australia. Most of the sampling areas were located within a 1- to 10-kilometer radius from agricultural grazing areas (Table 1, Figure 1A). Samples were generally collected during field necropsies in cooler winter months (Table 1, Figure 1B), when most culling and hunting occurs in Australia. Blood was drawn from the jugular vein and/or the heart and thoracic cavity, and collected in tubes (Becton Dickinson, Franklin Lakes, NJ, USA) with and without anticoagulant (EDTA) to obtain plasma and serum, respectively. Collection tubes were immediately refrigerated and transported to the laboratory. At the laboratory, serum and plasma were separated by centrifugation (10 min at 2000 g), transferred to 1.5 mL screw capped vials and stored at −80 °C until assayed.

### 2.2. Serological Methods

Serum and plasma were tested for *Pestivirus* antigen (Ag) and antibodies (Ab) against BoHV-1 and *Pestivirus* using the commercially available immunoenzymatic assay (ELISA) kits SERELISA^®^ BVD p80 Ag Mono Indirect (Synbiotics SAS, Lyon, France), SERELISA BHV-1 Ab Mono Blocking ELISA (Synbiotics SAS, Lyon, France) and the PrioCHECK™ BVD & BD p80 Serum Kit (ThermoFisher Scientific, Rockford, USA) according to the manufacturer’s instructions. For BoHV-1, samples with a ratio OD (optical density) sample/OD negative control ≤0.5 were classified as positive. This kit presents sensitivity (sn) of 96% and specificity (sp) 99%, according with the manufacturer. The *Pestivirus* kit detects antibodies targeted against the protein (p80/125), common to all BVDV and border disease virus (BDV) strains, with a manufacturer reported 97% sn and 98% sp for BVDV and 96% sn and 100% sp for BDV. Inhibition percentage (%INH) for each sample was calculated according to the manufacturer’s kit insert.

Samples with a percentage inhibition (%INH) of <50 were classified as negative, those with 50 ≤ %INH < 80 as weak positive, and %INH ≥ 80 as strong positive. Results for the *Pestivirus* antigen detection kit are expressed as an index = 0.5 × OD sample−OD Positive control (P). Any sample having an index ≥(−0.15 × OD P) was considered positive, <(−0.3 × OD P) was considered negative and between (−0.15 × OD P) and (−0.3 × OD P) was considered doubtful according to the manufacturer’s instructions.

Positive and negative controls were included in each run following the manufacturer’s recommendations. Furthermore, all deer samples were initially tested in pools of three, with all serum samples in positive pools being additionally sampled individually and in duplicate. Optical density was measured using a plate reader (ClarioStar—BMG Labtech, Ortenberg, Germany) at 450 nm wavelength.

### 2.3. RNA Extraction and RT-PCR

Due to the large number of animals sampled, only a subset of 144 sera was selected across all sampled regions to be screened by PCR (Table 1) for four agriculturally relevant viruses (EHDV, BEFV, *Pestivirus* and Simbu serogroup). These included all the samples with ELISA-Ag positive and doubtful results. RNA was extracted from 140 µL of serum or cell culture supernatant (positive controls) using a QIAamp^®^ Viral RNA Mini Kit (Qiagen, Valencia, CA, USA), according to the manufacturer’s instructions. Viral RNA was reverse transcribed using a Tetro cDNA Synthesis Kit (Bioline, London, UK) using random hexamers according to the manufacturer’s directions. RNA extracted from in vitro cultures for Akabane Virus, BEFV, EHDV and one bovine serum sample confirmed to be positive for BVDV, were used as positive controls. All culture material and BVDV positive sera were kindly donated by the Department of Jobs, Precincts and Regions, Victoria. PCR amplification was performed in a 25 µL reaction mixture containing 1× Green GoTaq Flexi buffer, 2 mM of MgCl_2_, 10 mM of dNTPs, 0.2 µM of both forward and reverse primers (Table 2), 0.625 units of GoTaq G2 DNA polymerase (Promega, Madison, WI, USA) and 1 µL of total genomic DNA template. PCR primers were obtained from the literature for the four viruses included in this study (Table 2 and references therein). Amplification was carried out in a T100 thermal cycler (BioRad, Hercules, CA, USA), and amplification products visualized by gel electrophoresis, using 2% agarose gel, RedSafe™ (iNtRON Biotechnology, Gyeonggi-do, Korea), and a high-resolution imaging system—ChemiDoc™ MP Imaging System (Bio-Rad, Hercules, CA, USA).

### 2.4. Statistical Analysis

Samples were categorized as “positive” or “negative” based on the results of the ELISA-Ab. Seroprevalence was calculated from the proportion of seropositive results of those tested and is presented with 95% confidence interval (CI), calculated using the Wilson score interval (www.epitools.ausvet.com.au). Binary logistic regression models were used to evaluate the effect of the sex, age category and sampling site on the antibody status. Logistic regression was performed using R version 4.0.0 (R Development Core Team, Vienna, Austria). Due to the sparse nature of the data, we performed the logistic regression analysis only on fallow deer (in which most of the positive samples were detected). Lastly, we used the two-tailed Fisher’s exact test to evaluate the hypothesis of non-random distribution of positive samples between fallow and rusa deer. *p* < 0.05 was considered statistically significant.

## 3. Results

### 3.1. Deer Sampling and Distribution

During the sampling period, 432 wild deer were sampled encompassing four deer species (200 fallow deer, 110 chital deer (*Axis axis*), 80 rusa deer and 42 sambar deer (*Rusa unicolor*)) across eastern Australia (Table 1). Sampling was conducted from November 2017 to November 2019, with most samples (72%) collected between June and October (Table 1, Figure 1B). Slightly more females (*n* = 229) than males (*n* = 196) were sampled, while no information was available for seven animals (Table 1, Figure 1D). Individuals were classified in three age categories based on morphological characteristics, including body size, tooth wear and antler growth: fawn (<1 year), yearling (1 to <2 years) and adult (≥2 years). Most of the samples came from adult individuals (*n* = 305), followed by yearlings (*n* = 103) and fawns (*n* = 17). Information on age was not available for seven animals (Table 1, Figure 1C).

### 3.2. ELISA Testing

**Figure 2:**
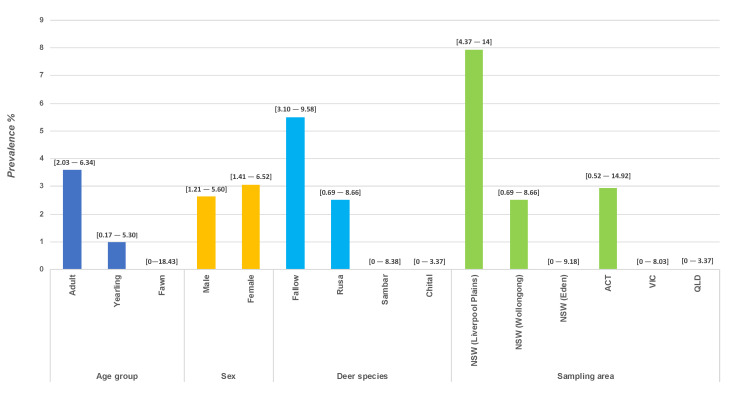
Prevalence of *Pestivirus* Antibodies: antibody prevalence was determined by ELISA and is represented as prevalence across age group, sex, species and sampling area. The 95% confidence interval is shown in brackets.

Sera and plasma samples from all 432 wild deer were screened by ELISA-Ab for *Pestivirus* and BoHV-1 (Table 1). All samples were negative for BoHV-1 antibodies. However, a total of 13 wild deer reacted positive for *Pestivirus* antibodies, resulting in an overall seroprevalence of 3.0%. Of the *Pestivirus* seropositive deer, 46.2% were sampled in June and 17.8% in August. Of the positive samples, 85% were obtained from adults. Additionally, all *Pestivirus* positive samples were collected from fallow and rusa deer, with a 5.5% and a 2.5% *Pestivirus*-seropositivity for each species, respectively (Figure 2).

The animals positive for *Pestivirus* ELISA-Ab were sampled in Australian Capital Territory (ACT; 7.7%) and New South Wales (NSW; 92.3%). Two out of the three sampling areas from this last state showed seropositive deer with a local prevalence of 7.94% in central NSW (Liverpool Plains), and 2.5% in coastal NSW (Wollongong) (Figure 2). Five of the 13 wild deer reacted as strong positives with test values (%INH) ranging between 80–92%; the remaining eight wild deer were weak positives with percentages ranging between 51–79% (Table 3).

Additionally, 321 out the 432 wild deer were also tested for *Pestivirus* antigens (see Table 1). Of the 321 deer tested, 278 (86.6%) showed a negative result, 24 (7.5%) revealed a doubtful result and 19 (5.9%) reacted positive. Additionally, only two of these positives overlapped with positive results shown for the ELISA-Ab screen.

Due to quasi-complete separation (i.e., most of the positives were in one category), we initially limited the predictors to the main effect of sex and age category (adult, yearling and fawn) or sex and location, and then tried to further simplify the model by considering sex only. In all models, the effect of the variables was not significant (*p* > 0.05). The proportion of seropositive *Pestivirus* results for fallow and rusa deer was similar (*p* = 0.36).

### 3.3. RT-PCR Screening

From the whole biobank of sera, 144 samples (see Table 1) were selected to be tested for *Pestivirus*, EDHV, BEFV and Simbu serogroup using previously validated RT-PCR primers sets [11,12,13,14]. These included all samples that had a doubtful or positive reaction for ELISA-Ag. Samples with hemolysis and insufficient volume were not included in the screening. No positive PCR amplification was obtained for any of the viruses screened, however, a positive result was obtained for all positive control samples in all runs.

## 4. Discussion

Australia’s wild deer populations have increased in abundance and distribution during recent decades, and the close interaction between deer and livestock is a risk for pathogen transmission. However, little is known about the epidemiological role of wild deer in Australia. This study constitutes the largest number of deer, and deer species, sampled in Australia to date for viral pathogens, and complements a recent study performed on a similarly large number of animals across multiple geographic locations for the presence of parasitic infections [15]. Moreover, this study indicates exposure of deer to *Pestivirus* and is the first report of antibodies to ruminant *Pestivirus*es in rusa deer. This baseline information is of value for monitoring the status of endemic livestock pathogens in Australian deer, and for evaluating the risk of disease transmission between wild deer and livestock.

Although numerous pathogens have been detected in different cervids worldwide, including agriculturally relevant viruses [2], there is scarce knowledge about the viral infection status of Australian wild deer populations, with only four previous serosurveys being performed on red, fallow and rusa deer, but limited in their geographical coverage [7,8,9,10]. In contrast, the present study is based on the assessment of large sample sizes from four deer species collected throughout eastern Australia.

Our findings reveal the presence of antibodies in Australian wild deer species, against ruminant *Pestiviruses* (BVDV and BDV), which infect a variety of wild and domestic ungulate species and are associated with severe economic losses in livestock production worldwide [16]. In this study, positive serologic reactions were recorded in fallow and rusa deer at similar rates. Although the serosurveys have been proven to be a fundamental tool when it comes to disease surveillance, serological testing used in this study cannot distinguish between BVDV and BDV infections.

To our knowledge, this is the first report of *Pestivirus* antibodies in rusa deer. Additionally, in accordance with our findings, *Pestivirus* antibodies were identified previously in Australian fallow deer [7,8]. Seropositive fallow deer were first reported by Munday et al. (1972) [7] in Tasmania, with a prevalence of 14.5%. Ten years later, another serosurvey described a prevalence of 1.2% (1/86) in fallow deer sourced from New South Wales [8], in an area also included in our study. Moreover, McKenzie et al. [9] reported a prevalence of 4% in red deer sourced from 20 localities in eastern Australia. In comparison with the previous report for fallow deer from New South Wales [8], a higher seroprevalence is reported in the present study. The use of a different detection assay could have influenced the prevalence obtained. Compared with previous Australian serosurveys that used a virus neutralization test targeting only BVDV1, we used a more robust immunoassay that could detect antibodies against BVDV1, BVDV2 and BDV. The higher seroprevalence in this study might also reflect a real change in prevalence in the last 40 years, possibly due to an increase in deer density leading to greater interaction with livestock. Although samples from red deer were not included in our analyses, seroprevalence detected in fallow and rusa deer is comparable to that described in red deer by McKenzie et al. [9]. These findings may indicate that red, fallow and rusa deer are similarly exposed to *Pestivirus*.

Fallow deer, chital deer and, to a lesser extent, rusa deer, are gregarious, and hence, their transmission of pathogens would be expected to be higher compared to sambar deer, which are usually solitary [17]. No seroprevalence for *Pestivirus* was observed for sambar deer in this study, however, sampling numbers were low in comparison to other species. The sampling location for sambar deer in this study was composed of forested areas within mountain landscapes mostly distant from livestock grazing areas; however, the chital deer samples were collected from within pastoral areas where they are known to interact with cattle.

There is conflicting evidence in the literature surrounding prevalence of *Pestivirus* in cervids and known contact with cattle. Studies [18,19,20] have reported that close contact with cattle can induce high seroprevalence in wild cervids; however, a study by Frolich et al. [21] contrasted these findings and hypothesized an independent cycle as responsible for intrapopulation persistence. Additionally, the identification of persistently infected mule deer (*Odocoileus hemionus*) [22] and white-tailed deer (*Odocoileus virginianus*) [23,24,25] suggests that BVDV can sustain itself in deer populations without contact with cattle. The susceptibility of sambar and chital deer to BVDV has not been demonstrated, and we cannot completely discount the lack of susceptibility as a possible cause of these negative results. However, it is possible that mortality of persistently infected animals or differences in social behavior between chital and sambar deer with the other deer species, which could also explain these negative results.

Most of the seropositive *Pestivirus* outcomes were recorded in adult deer, which is not unexpected, as they constituted ~70% of the animals sampled in this study. Although antibody levels could be detected in the sampled deer, it is not possible to know when the animals were exposed to the virus. Seroconversion in cattle usually appears two weeks after infection, with the titer continuing to rise for 10 to 12 weeks; after this time, a plateau is reached [26,27]. Similarly, deer experimentally infected with BVDV exhibit a similar seroconversion course, developing antibodies at 8 to 15 days post infection [28,29]. However, antibodies against BVDV have been demonstrated to remain in serum for longer than three years [30].

*Pestivirus* antibody detection was performed in serum and plasma using a blocking ELISA kit with sensitivity and specificity comparable with other similar kits [31], also validated for non-bovine samples [32,33,34]. Concordance in results were found in eight overlapped specimens (serum and plasma), and two of the remaining overlapped specimens revealed positive outcomes only in one of the samples. Although this discrepancy is not enough to state which sample is better for *Pestivirus* antibody detection in deer, previous studies did not find variation between serum and plasma for viral antibody detection [35,36,37]. It is possible that other factors including the quality of the sample and antibody concentration could have affected the results in some samples.

Overall, the seroprevalence for *Pestivirus*es was 3%, considerably lower than the 52.6% observed in Australian cattle [16]. Similar findings were detected in European countries during serosurveys of *Pestivirus*es in deer to evaluate the epidemiological importance of deer in BVDV eradication programs: Belgium, 1.3% [38], Germany, 2% [39], Switzerland, 2.7% [3], and Italy, 4.5% [40]. The authors concluded that despite regular interactions with farmed ruminants, infection in deer was occasional with virus transmission from cattle to deer, and therefore, the possibility of deer being a source of infection for cattle was remote.

In contrast to other reports, we did not detect any infected deer by PCR, although the RT-PCR assay we used detects a broad range of *Pestivirus*es from pigs, cattle and sheep [11]. Thus, based on detection of a low seroprevalence for ruminant *Pestivirus*es in the deer population studied, the fact that we did not identify any persistently infected (virus positive, antibodies negative) deer, and given the high number of seropositive cattle in Australia, we consider it more probable that deer are an accidental spillover host rather than a reservoir host for ruminant *Pestivirus*es, and that persistently infected cattle could transmit these viruses to wild deer.

*Pestivirus* antigen detection by ELISA resulted in 5.9% and 7.5% of samples testing positive and doubtful, respectively. However, RT-PCR negative results were obtained in all the samples. The ELISA kit utilized is reported to detect *Pestivirus* antigens in ruminant samples, however, there is no published information about validation with cervid samples. A similar antigen detection methodology was previously used for roe deer (*Capreolus capreolus*) [41], with positive results obtained. However, those findings were not confirmed by RT-PCR. A possible explanation for the false positive antigen results could be variations in the primer target region not detectable by RT-PCR used. A second explanation is that there were unspecific cross-reactions, but further work is needed to determine the exact cause of the potential false positive antigen ELISA results obtained in this study.

We acknowledge that a limitation of this study is the type of tissue utilized for detection of BVDV genetic material. Although there are studies that used serum to detect BVDV by PCR [42,43], BVDV has trophism for epithelia of both the alimentary and integumentary systems [44], however, due to the collection strategies available to us during the sampling procedures, specimens that would be more reliable in detecting low levels of viral nucleic acid over a longer period of infection time, such as reproductive tissues [45,46] or spleen [47], were not available for testing.

BoHV-1 is the best characterized member of the subfamily *Alphaherpesvirus*, responsible for Infectious Bovine Rhinotracheitis (IBR), a cattle disease of major economic concern worldwide [48], and widespread in Australian cattle with a seroprevalence of 25–40% [49]. Susceptibility to BoHV-1 of wild cervids has previously been demonstrated [5,50], and additionally, several ruminant *Alphaherpesviruses* related to BoHV-1 have been isolated from cervids, including cervid herpesvirus 1 (CvHV-1) in red deer [51] and cervid herpesvirus 2 (CvHV-2) in reindeer (*Rangifer tarandus*) [52]. Our failure to detect antibodies against BoHV-1 is consistent with previous reports in Australia, which demonstrated an absence of BoHV-1 antibodies in fallow deer in Tasmania [7] and red deer in Queensland [9]. Furthermore, we can state that there is no evidence of cervid alphaherpesviruses in Australian wild deer, since serological cross reactivity by both virus neutralization and ELISA between ruminant alphaherpesviruses, including CvHV-1 and -2, is well documented [53,54,55].

The assessment of a subset of 144 serum samples by PCR also revealed no evidence of acute infection for the other viral livestock pathogens screened in this study. BEFV, EHDV and Akabane virus (a member of the Simbu serogroup) are endemic in Queensland and they have a seasonal spread in New South Wales [56,57,58]. Moreover, these viruses remain undetected in Victoria [57,58]. As they are vector-borne viruses, their occurrence is limited by the effect of cold weather, which restricts the distribution of their vectors. Previous studies performed in Australian deer reported serological evidence for BEFV, EHDV and Akabane virus in red deer from south-eastern Queensland [9]. Moreover, Moriarty et al. [10] found seropositive outcomes in a small sample of rusa deer in coastal central NSW for Akabane virus and EHDV. All the samples screened in the present study were negative for these viruses; however, the presence of vector species and previous evidence highlights the need for further serologic analysis to determine the role of deer as a spillover or reservoir host for these viruses, particularly in Queensland and New South Wales where BEFV, EHDV and Akabane virus are endemic. One limitation of this study was that all deer were sampled in the colder winter months, which would also lessen the activity of the vectors necessary for virus transmission.

## 5. Conclusions

Our findings provide an overview of the current *Pestivirus* infection status of wild deer in eastern Australia. The low prevalence of *Pestivirus* antibodies and negative findings for the viruses tested suggests that wild deer are an incidental spill-over host, and not a reservoir host. However, considering the substantial increase observed in fallow deer seroprevalence compared with a previous report [8], and the expected increase in distribution and abundance [1] (in the absence of substantial control), we cannot rule out the possibility that deer species sampled in this study could be a future source of infection for livestock.

## Figures and Tables

**Table 1:** Population characteristics and distribution of deer sampled and tested in this study.

States or Territory	Animals	Sampling Location	Species	Sex			Age Groups				Month of Sampling									No. Deer Tested by		
				M	F	N.r.	Ad	Yrl	Fw	N.r.	Feb	Mar	Apr	May	Jun	Jul	Aug	Oct	Nov	ELISA Ab	ELISA Ag	PCR
NSW	244	Liverpool Plains *	Fallow	74	52	0	74	47	5	0	0	0	0	0	39	0	87	0	0	126	126	42
		Eden *		12	21	5	21	9	3	5	0	0	0	0	0	0	18	15	5	38	0	18
		Wollongong *	Rusa	69	11	0	68	12	0	0	43	0	14	0	13	0	0	10	0	80	66	7
ACT	34	Canberra *	Fallow	14	20	0	26	8	0	0	0	0	0	0	34	0	0	0	0	34	0	10
VIC	44	Alpine National Park	Sambar	17	14	1	19	6	6	1	10	0	15	7	0	0	0	0	0	32	16	17
		Upper Yarra Flats		2	6	1	6	1	1	1	0	0	0	0	8	0	0	1	0	9	8	4
		Yellinbo		0	1	0	0	0	1	0	0	0	0	0	0	1	0	0	0	1	0	1
			Fallow	0	2	0	1	0	1	0	0	0	0	0	0	2	0	0	0	2	0	2
QLD	110	North east Queensland *	Chital	41	69	0	90	20	0	0	0	47	0	0	5	0	0	0	58	110	105	43
Total	432			229	196	7	305	103	17	7	53	47	29	7	99	3	105	26	63	432	321	144

* Sampling was conducted in areas close to livestock farms; NSW: New South Wales; ACT: Australian Capital Territory; VIC: Victoria; QLD: Queensland; F: female, M: male, Ad: adult, Yrl: yearling, Fw: fawn, N.r.: Not recorded; chital deer (*Axis axis*), rusa deer (*Rusa timorensis*), sambar deer (*Rusa unicolor*), fallow deer (*Dama dama*).

**Table 2:** List of oligonucleotides and PCR conditions used in this study.

Virus	Target Region	Primer Name	Sequence 5′–3′	Amplicon Length (bp)	PCR Condition	Reference
*Pestivirus*	5′UTR	324	ATGCCCWTAGTAGGACTAGCA	288	95 °C × 2 min 40 cycles (95 °C × 45 s, 52 °C × 45 s, 72 °C × 45 s) 72 °C × 5 min	[11]
		326	WCAACTCCATGTGCCATGTAC			
Simbu Serogroup	Segment S	Uni-S-59F	GATGWCCWCAACGGAAT	215	95 °C × 2 min 40 cycles (95 °C × 45 s, 55 °C × 45 s, 72 °C × 45 s) 72 °C × 5 min	[12]
		Uni-S-254R	TGGGGAAAATGGTTATTAAC			
BEFV	Glucoprotein G	GF	ATGTTCAAGGTCCTCATAATTACC	1871	95 °C × 2 min 40 cycles (95°C × 45 s, 52 °C × 45 s, 72 °C × 2 min) 72 °C × 5 min	[13]
		GR	TAATGATCAAAGAACCTATCATCA			
EHDV	NS3	NS3F	CAGCGCYWTATWCGATATTG	533	95 °C × 2 min 40 cycles (95 °C × 45 s, 55 °C × 45 s, 72 °C × 60 s) 72 °C × 5 min	[14]
		NS3R	TCCGGAGATACCTCCATTAC			

**Table 3:** Description of deer samples that tested positive for *Pestivirus* antibodies.

Sample	Species	Sampling Month	Sex	Age	Location	Anti-*Pestivirus* ELISA			
						Serum		Plasma	
						Result	%INH ^a^	Result	%INH ^a^
1	Fallow	June	F	Ad	New South Wales	WP	65	Neg	-
2	Fallow	June	F	Ad	New South Wales	SP	91.9	SP	91.4
3	Fallow	June	F	Ad	New South Wales	WP	73.7	WP	57.4
4	Fallow	June	F	Yrl	New South Wales	WP	73.6	WP	59.0
5	Fallow	June	F	Ad	New South Wales	WP	61.6	WP	67.0
6	Fallow	August	M	Ad	New South Wales	WP	71.6	WP	67.1
7	Fallow	August	M	Ad	New South Wales	SP	80.1	WP	79.4
8	Fallow	August	M	Ad	New South Wales	SP	84.4	SP	83.3
9	Fallow	August	M	Ad	New South Wales	SP	87.2	SP	80.1
10	Fallow	August	M	Ad	New South Wales	SP	87.2	WP	78.9
11	Rusa	February	ND	ND	New South Wales	WP	51.8	NS	-
12	Rusa	October	F	Ad	New South Wales	Neg	-	WP	60.1
13	Fallow	June	M	Ad	Australian Capital Territory	NS	-	WP	56.4

^a^ %INH: percentage of inhibition obtained by ELISA; ND: no data, F: female, M: male, Ad: adult, Yrl: yearling; WP: weak positive, SP: strong positive, NS: no sample, Neg: negative.
